# Saunders’s Medical Hand-Atlases

**Published:** 1902-03

**Authors:** 


SAUNDERS'S MEDICAL HAND-ATLASES. Atlas and Epitomé of Special Pathologic Histology. By DOCENT DR. HERMAN BÜRCK, of the Pathologic Institute of Munich. Edited by Ludvig Hektoen, M. D., Professor of Pathology in Rush Medical College, Chicago. Vol. II.—Liver; Urinary Organs; Sexual Organs; Nervous System; Skin; Muscles; Bones. With 123 colored illustrations on 60 lithographic plates and 192 pages of text. Philadelphia and London: W. B. Saunders & Co. 1901. [Cloth, $3.00 net.]

This is the second of the three volumes on pathologic histology promised in the prospectus of the Saunders-series of medical hand atlases, and it is a most excellent work in which the standard set in the first volume has been maintained. The special subjects covered are the liver, the urinary organs, sexual organs, nervous system, skin, muscles and bones. There are 123 colored illustrations on 60 lithographic plates, and as in the previous volume these add to the distinct value of the book as a scientific work of art. The third volume which soon will be issued, and which will complete the pathologic series, will treat of general pathology. N.W.W.

